# Establishment of the Nichols strain as the type strain of Treponema pallidum

**DOI:** 10.1099/ijsem.0.006697

**Published:** 2025-02-27

**Authors:** Steven J. Norris

**Affiliations:** 1 Department of Pathology and Laboratory Medicine, McGovern Medical School, University of Texas Health Sciences Center at Houston, Houston, Texas, USA; 2 Department of Microbiology and Molecular Genetics, McGovern Medical School, University of Texas Health Sciences Center at Houston, Houston, Texas, USA

**Keywords:** bejel, Nichols strain, syphilis, *Treponema pallidum*, type strain, yaws

## Abstract

In this article, it is proposed that the Nichols strain of *Treponema pallidum* be established as the type strain. *T. pallidum* was first identified as the causative agent of syphilis in 1905, and the Nichols strain was isolated in 1912 by inoculation of a rabbit with cerebrospinal fluid from a patient with neurosyphilis. The Nichols strain has been maintained by serial passage in rabbits for over a century, and historically most studies of *T. pallidum* have been conducted using this strain. In recent years, a procedure for continuous *in vitro* culture of *T. pallidum* in a tissue culture system has been developed, making propagation of this spirochaete easier and hence facilitating research. The Nichols strain has >99% DNA homology with a group of organisms that cause syphilis, bejel/endemic syphilis and yaws in humans, a yaws-like disease in primates and spirochaetosis in rabbits and hares. This group is highly similar in terms of their gene and G+C content, genome synteny, cell morphology, natural dependence on mammalian hosts and ability to cause long-term infections; variation occurs in host range, modes of transmission, aptitude for dissemination, manifestations, congenital infection and geographical distribution. Availability of a type strain will aid in the formal acceptance of *T. pallidum* subspecies first described in 1984 and supported by recent whole-genome analyses of numerous strains from the *T. pallidum*-related group.

Drs Fritz Schaudinn and Erich Hoffman [1] first described the causative agent of syphilis in 1905, characterizing the organism as a slender spirochaete with a length of 4–10 µm, a width less than 0.5 µm and 3–12 turns in its helical shape. Its characteristic motility, consisting of rotation, reversal and flexion, was also noted in this earliest portrayal. The bacterium was first named *Spirochaeta pallida*, but in the same year, Schaudinn recognized its differences from previously characterized *Spirochaeta* species and changed the name to *Treponema pallidum* [2]. Although the identification of *T. pallidum* as the cause of syphilis was questioned initially, by 1906, over 200 articles had been published confirming this finding (reviewed by Buschke and Fischer [3]). The study of * T. pallidum* progressed rapidly during the initial decade of the 20th century, including the application of darkfield microscopy by Richter, Landsteiner and others to improve visualization of the spirochaete (as reviewed in [4]), the identification of Compound 606 (the arsphenamine Salvarsan, later refined into the less toxic derivative Neosalvarsan) as a treatment for syphilis by Ehrlich and colleagues [5,6] and the discovery of antibody reactivities useful in the diagnosis of syphilis by von Wassermann and other investigators [7]. Many early studies were focused on *in vitro* isolation and culture of *T. pallidum*, and success was reported as early as 1906 [8,9]. Culture methods included the combined use of tissue fragments, serum and anaerobic conditions, and indeed, some of these experiments may have been successful. However, the *T. pallidum* cultivation procedures were found to be either irreproducible or the result of contamination with commensal skin spirochaetes (such as *Treponema phagedenis*) [10,11].

Physiologic studies and cultivation efforts continued through the decades [9,12,16]. In 1981, Fieldsteel *et al*. [17] reported limited, but highly reproducible, multiplication of *T. pallidum* in a coculture system with Sf1Ep rabbit epithelial cells under microaerobic conditions. Additional studies have shown that long-term *T. pallidum* survival, metabolic activities and propagation are dependent upon the presence of ~1.5–5% oxygen [13,16,18], thus establishing that the organism is a microaerophile. Further refinement of this system in 2017 through the utilization of a more complex basal medium resulted in continuous *in vitro* culture of several *T. pallidum* strains [25,27], including both syphilis and bejel (endemic syphilis) isolates. Thus far, *in vitro* culture of yaws isolates has been unsuccessful. The availability of a system for *in vitro* cultivation has provided new avenues for *T. pallidum* research, including more detailed analyses of its growth requirements [25], host–pathogen interactions [28], antimicrobial susceptibility [29,34], genetics [35], gene expression [36], protein synthesis [37] and gene function through genetic manipulation [38,42].

## The *T. pallidum*-related group

The spirochaetes that cause syphilis, yaws, bejel and infections in rabbits and hares are all very closely related and are hereafter called the *T. pallidum*-related group. These organisms are morphologically indistinguishable, are all obligate parasites of humans or other mammals and cause persistent infections with varying degrees of tissue invasion. Strains of *T. pallidum* that cause syphilis in humans are the most invasive organisms of this group [43,47]. The disease is sexually transmitted, and initial infections are characterized by the formation of a localized lesion called a chancre at the site of exposure within 2–4 weeks. These lesions contain large numbers of *T. pallidum* that can be detected during the active phase using darkfield microscopy, immunohistochemistry, immunofluorescent microscopy or nucleic acid-based tests. The organisms readily disseminate through the circulatory system and can colonize any tissue. Within weeks to months after infection, multiple secondary lesions (often presenting as a rash) may occur in the skin or mucus membranes. If not treated with penicillin or other antibiotics, the infection enters into a latent phase which may continue for the lifetime of the patient. In roughly one-third of untreated patients, late (or tertiary) manifestations occur, affecting the nervous system (neurosyphilis) or the heart and major blood vessels (cardiovascular syphilis). A third form of late syphilis is called gummatous syphilis and is characterized by the formation of destructive granulomatous lesions (gummas) at any location. Finally, congenital syphilis results from the passage of *T. pallidum* from the mother to the foetus, causing either stillbirth, active infection at the time of birth or latent infection which can then progress to symptomatic disease later in childhood [44,48, 49].

Another organism of this group, initially described in 1905 as ‘*Spirochaeta pertenuis*’ by Castellani [50] and later called *Treponema pertenue* [51], causes yaws. The human infection with this spirochaete occurs in tropical areas and is spread by skin-to-skin contact, typically within the childhood and adolescent years [52]. Although it often causes only skin lesions, yaws can result in painful, destructive lesions of the nose or of the arm or leg bones; long-term lesions can cause disfigurement, including a bowing of the lower legs called sabre shins. A naturally occurring disease of primates (e.g. baboons and chimpanzees) is caused by organisms that are genetically indistinguishable from those that give rise to yaws in humans [53,54]; in primates, this disease can be transmitted sexually and may result in severe lesions in the genital region [54]. Endemic syphilis, now commonly referred to by the Arabic term bejel, is also transmitted by skin-to-skin contact among children but is found in arid climates; it also causes skin lesions and disfiguring destruction of cartilage or bone. Both yaws and bejel can cause lifetime infections if not treated with antibiotics, but congenital transmission of these diseases does not appear to occur [52]. The differences in dissemination, the occurrence of internal lesions and congenital transmission may be related to varying temperature sensitivity [55,56] and minor genetic differences between the subgroups of *T. pallidum* causing syphilis, yaws and bejel (see below). Another member of the *T. pallidum*-related group, *Treponema carateum*, is described as the causative agent of pinta, an infection that occurred in some areas of South America [52]; pinta cases have not been reported in recent years. *T. carateum* was detected by microscopy but has never been isolated or otherwise characterized. *Treponema paraluiscuniculi*, or the proposed species *Treponema paraluisleporidarum* [57,58], cause infections in lagomorphs (rabbits and hares) and also share characteristics of the *T. pallidum*-related group [59].

## Nomenclature history

In the 1984 edition of *Bergey’s Manual of Systematic Bacteriology*, Dr Robert M. Smibert established subspecies of *T. pallidum* [60]. Prior to that edition, the causative agents of syphilis and yaws were called *T. pallidum* and *T. pertenue*, respectively. There was no separate designation for the agent of bejel, which was considered to be a subset of *T. pallidum*. In 1980, Miao and Fieldsteel [61] utilized DNA–DNA reassociation hybridization to determine that the *T. pallidum* strains Nichols and KKJ and the *T. pertenue* strain Gauthier had >98% overall sequence identity. In contrast, virtually no hybridization was observed between the DNA of the pathogenic treponemes with that of the nonpathogenic *Treponema* species *T. phagedenis* and *Treponema refringens* [62]. Based on their (1) high degree of sequence identity indicated by DNA–DNA hybridization; (2) near identical G+C content (in the range of 52.4–53.7% based on melting temperature); (3) indistinguishability of cells at the morphologic level; (4) extreme dependence on the human host and (5) similar disease manifestations, Dr Smibert decided that listing *T. pallidum* and *T. pertenue* as separate species was not justified. He therefore renamed them as the subspecies *T. pallidum* subsp. *pallidum* and *T. pallidum* subsp. *pertenue*. In addition, he established a third subspecies, * T. pallidum* subsp. *endemicum*, as the causative agent of bejel. *T. carateum* was left as a separate species because of the lack of DNA–DNA hybridization or mol% G+C data.

The genomes of members of the *T. pallidum*-related group consist of a single circular chromosome of ~1.14 Mb, and over 500 genomes of *T. pallidum* strains are currently available in the U.S. National Center of Biotechnology GenBank database. No other replicons have been identified in *T. pallidum* genomic sequences. Representative complete genomes have between 1089 and 1135 annotated genes, with near complete synteny [63]; the main differences are typically single-gene insertions or deletions, with many of these being members of the *T. pallidum* Repeat (*tpr*) gene family. The overall nucleotide sequence identity across the three subspecies is more than 99.7% [64], thus justifying their grouping as a single species. The only other closely related species identified to date are *T. paraluiscuniculi* and the proposed *T. paraluisleporidarum*, which have an overall sequence identity with *T. pallidum* of ~99.3% [65].

Although based initially on different disease manifestations, transmission patterns and geographic distributions, the subspecies designations described by Smibert [60] have withstood the test of time and the flood of genetic data over the past four decades. Despite 99.7% overall nucleotide sequence identity, the *T. pallidum* subspecies do form separate entities based on genetic differences, as well as pathogenic patterns. Phylogenetic trees based on whole-genome comparisons consistently feature longer branch lengths among the three subspecies, yielding three distinct groups; in addition, within the *T. pallidum* subsp. *pallidum* group, there are two clusters (called the Nichols and SS14 clusters, named for two representative strains) that have resulted from a more recent divergence [63,66,71]. The differences between the subspecies and the clusters are related to the occurrence of nonsynonymous changes in a subset of genes, as described in the above references.

The small group of *T. pallidum*-related spirochaetes have certain shared ‘treponeme-specific’ genes [e.g. the cytoplasmic filament protein A gene (*cfpA*)] and significant overall protein sequence similarity with other species within the genus *Treponema*. In addition, many of the *Treponema* species are also host-dependent and are found in nature only in association with mammalian hosts (usually in the oral cavity, intestinal tract or integument). However, the *T. pallidum*-related group is distinct from all other *Treponema* species characterized to date in terms of their extremely small genome size, overall DNA sequence identity and much higher G+C content [72]. A phylogenomic tree based on the concatenated protein products from 120 single-copy genes [73] shows that *T. pallidum* is well separated from most *Treponema* species, although *T. phagedenis*, *Treponema medium* and *Treponema vincentii* retain some degree of similarity. These differences indicate that the *T. pallidum*-related group has diverged substantially from other *Treponema* species in the process of adapting to the mammalian host tissue environment.

Overall, there is overwhelming evidence for the existence of three subgroups within *T. pallidum* which, on a biological and genetic basis, should be considered subspecies. Indeed, the subspecies designations have been used in over 300 scientific journal articles since 1984. However, these designations are not considered validly published. Therefore, the *T. pallidum* subspecies are not included in the List of Prokaryotic names with Standing in the Nomenclature (https://LPSN.dsmz.de) and thus cannot be utilized in the *Bergey’s Manual of Systematics of Archaea and Bacteria*.

## The Nichols strain as type strain

The main purpose of this article is to establish the Nichols strain as the type strain for *T. pallidum* as a first step of the process to obtain valid publication of the three subspecies. The establishment of a type species is being carried out under the provisions of Rule 18f in the 2022 Revision of the International Code of Nomenclature of Prokaryotes [74], which states (in part) the following: ‘If a description or illustration constitutes, or a dead preserved specimen has been designated the type of a species [Rule 18a(1)] and later a strain of this species is cultivated, then the type strain may be designated by the person who isolated the strain or by a subsequent author.’ In this case, the type of the species *T. pallidum* was designated by the descriptions and illustrations produced by Schaudinn and Hoffman [1]. The Nichols strain was later isolated and propagated by rabbit inoculation by Nichols and Hough [75]. The article you are reading represents the designation of the Nichols strain as the type strain ‘by a subsequent author’.

The Nichols strain was isolated by Nichols and Hough [75] in 1912 using inoculation of a rabbit’s testes with cerebrospinal fluid (CSF) from a patient with recurrent neurosyphilis following treatment with salvarsan. The choice of rabbit testicular inoculation was based on prior reports (dating back to 1906) of successful transfer of the infection to rabbits by this means (reviewed by Willcox and Guthe [9]). Swelling of one of the testes and the presence of spirochaetes in an aspirate from the tissue were noted on day 50 after inoculation with 3 ml of CSF per testis, and the infection was successfully transferred to another rabbit on day 74. The strain was maintained by continuous serial passage in rabbits (and perhaps other animals, such as hamsters or guinea pigs [56]) until the 1970s. Thereafter, long-term storage of viable *T. pallidum* extracts by freezing at either −70 °C or under liquid N_2_ temperatures (with the addition of 10–20% glycerol as a cryoprotectant) became a common laboratory practice. The passage history of the Nichols strain is not clearly documented and likely involved many parallel pathways. Prominent participants in this process in the 1900s included the Nichols laboratory (until 1923) [76], the very active group of Brown and Pearce [77], Drs Turner, Hollander and Hardy and Ms Ellen Nell at the International Treponematoses Laboratory Center at Johns Hopkins University [56], Drs Ruth Boak and James N. Miller and the U.S. Centers for Disease Control and Prevention (CDC). More recently, the laboratories of Lorenzo Giacani, Sheila A. Lukehart, Michael V. Norgard, Steven J. Norris, Justin D. Radolf, David Šmajs and Caroline Cameron have been involved in maintaining the strain. The Nichols strain has recently been deposited at two repositories at BEI Resources and the Leibniz-Institut Deutsche Sammlung von Mikroorganismen und Zellkulturen (DSMZ) to provide more systematic preservation and access.

The selection of the Nichols strain as the type strain was based on its following properties: (1) The Nichols strain is the oldest available strain and was isolated within a few years of the initial identification of *T. pallidum* as the causative agent of syphilis. (2) It has been the most widely used strain for scientific studies both historically and in recent years. (3) It was the first *T. pallidum* strain for which a genomic sequence was available [78]. (4) It remains representative of *T. pallidum* genetic, physiologic, morphologic and pathogenic properties. As part of the latter aspect, *T. pallidum* Nichols has retained its infectivity in humans, as demonstrated in an (unethical) human inoculation study [79] and a case of accidental laboratory infection [80]. Another candidate for the type strain would be *T. pallidum* Street Strain 14 (also called SS14), which was isolated in 1977 from a patient in Atlanta, Georgia, USA, by a team that included staff members of the CDC. As indicated above, the Nichols and SS14 strains are considered to be representative of two major phylogenetic clusters of *T. pallidum* strains from human syphilis cases. Because of the factors listed above, *T. pallidum* Nichols was chosen as the type strain, but SS14 should be considered an additional reference strain for *T. pallidum*. As with all micro-organisms, microevolution is continuing in *T. pallidum*, as indicated by the recent selection of macrolide-resistant strains as well as the frequent discovery of new, novel genotypes [66,67, 69, 81,87]. Thus far, *T. pallidum* variants identified in venereal syphilis cases have been associated with either the Nichols- or SS14-associated phylogenetic clusters, with the exception of some cases of sexually transmitted infection with *T. pallidum* strains that have the genotype of bejel isolates [88].

It should be noted that both the Nichols and SS14 strains, although apparently of clonal origin, exhibit definite intrastrain heterogeneity [63,89], as substantiated by the isolation of Nichols strain clones with distinct genotypes [35]. This heterogeneity is limited to a small number of insertion–deletion events, single nucleotide variations, repeat sequence number variation and homopolymer tract length differences, apparently arising during passage in rabbits and, to some extent, during *in vitro* culture [35]. However, it is believed that the uncloned Nichols strain should serve as the type strain rather than an isolated clone, because the properties of any one chosen clone may not be representative of the entire population.

The Nichols strain can be propagated either by rabbit inoculation [90] or by *in vitro* culture [26,27]; for details regarding these procedures, refer to the cited articles. In its current form, the *in vitro* culture system requires Sf1Ep cottontail rabbit epithelial cells, a complex culture medium called TpCM-2, heat-inactivated foetal bovine serum and incubation in a low oxygen atmosphere (1.5% O_2_, 5% CO_2_, balance N_2_) at 34 °C [27]. The doubling time during *in vitro* culture is long (~40 h) but is similar to the estimated growth rate of 30–33 h occurring during rabbit infection [91,92]. Under standard conditions, cultures typically need to be passaged every 6–8 days [25,27].

The Nichols (Houston) strain was obtained from James N. Miller (University of California at Los Angeles) and has been maintained in the author’s laboratory at the University of Texas Health Science Center at Houston since 1982. The strain used for deposition in repositories was maintained by serial passage in rabbits with the frequent use of frozen stocks to minimize the number of passages required; the deposited strain has not been passaged *in vitro*. The Nichols (Houston) strain is available from BEI Resources (as Catalogue No. NR-59701) or from the DSMZ (as DSM 117211). The intrastrain heterogeneity present in Nichols (Houston) was described in detail by Edmondson *et al*. [35]. Other passages of the Nichols strain, such as Nichols (Seattle), are likely to be highly similar to Nichols (Houston) but may differ in the degree of intrastrain heterogeneity.

## Emended description of *Treponema pallidum* (Schaudinn and Hoffman 1905) Schaudinn 1905, 1728^AL^ (*Spirochaeta pallida* Schaudinn and Hoffman 1905, 528)

pal'li.dum. L. neut. adj. *pallidum*, pale, pallid.

Tightly coiled, ~0.18 µm in diameter by 6–20 µm in length. Spiral to wave-shaped with a uniform coil wavelength of ~1.1 μm and an amplitude of 0.2–0.3 μm. The ends of the cells are pointed, and a protrusion of the outer membrane at the end is often visible in well-preserved specimens by electron microscopy with negative staining. Two to four periplasmic flagella are inserted into each end of the cell and overlap in the middle of the cell. Cells are highly motile with rotational motion and the common occurrence of flexion and rotational reversal. Microaerophilic, with an optimal O_2_ concentration in the range of 1–5%.

Obligate pathogens of humans with subgroups causing syphilis, yaws and bejel (endemic syphilis). Sequence comparisons and DNA–DNA hybridization indicate that >99.7% DNA homology exists between *T. pallidum* strains, with distinct genotypes existing within the subgroups causing syphilis, yaws and bejel. Naturally occurring infections of other primates (including chimpanzees and baboons) occur and are caused by *T. pallidum* strains that are genetically indistinguishable from those causing yaws in humans. Experimental infection of rabbits, guinea pigs, hamsters and primates is possible and has been commonly used for the isolation of *T. pallidum* strains. Continuous *in vitro* propagation of *T. pallidum* strains causing syphilis and bejel has been achieved in a system utilizing co-incubation with a rabbit epithelial cell culture; strains causing yaws have not as yet been cultured *in vitro*.

*Source:* Human patients with syphilis, yaws or bejel; primates with a yaws-related infection.

*DNA G+C content (mol%):* 52.8 (genome sequence); 52.4–53.7 (Tm).

*Type strain:* Nichols.

*EMBL/GenBank accession number (16S rRNA gene):* M88726.

*EMBL/GenBank accession number (Nichols strain genome sequence):* NC_021490.

## Abbreviations

CDC: centers for disease control and prevention
CSF: cerebrospinal fluid
